# Shortening of low-flow duration over time was associated with improved outcomes of extracorporeal cardiopulmonary resuscitation in in-hospital cardiac arrest

**DOI:** 10.1186/s40560-020-00457-0

**Published:** 2020-06-15

**Authors:** Akiko Higashi, Taka-aki Nakada, Taro Imaeda, Ryuzo Abe, Koichiro Shinozaki, Shigeto Oda

**Affiliations:** 1 Department of Emergency and Critical Care Medicine, Chiba University Graduate School of Medicine, 1-8-1 Inohana, Chuo, Chiba, 260-8677 Japan; 2 The Feinstein Institute for Medical Research, Northwell Health, 350 Community Dr., Manhasset, New York, NY 11030 USA

**Keywords:** Rapid response system (RRS), Extracorporeal cardiopulmonary resuscitation (ECPR), Cardiac arrest, Low-flow duration (LFD)

## Abstract

**Introduction:**

Quality improvement in the administration of extracorporeal cardiopulmonary resuscitation (ECPR) over time and its association with low-flow duration (LFD) and outcomes of cardiac arrest (CA) have been insufficiently investigated. In this study, we hypothesized that quality improvement in efforts to shorten the duration of initiating ECPR had decreased LFD over the last 15 years of experience at an academic tertiary care hospital, which in turn improved the outcomes of in-hospital CA (IHCA).

**Methods:**

This was a single-center retrospective observational study of ECPR patients between January 2003 and December 2017. A rapid response system (RRS) and an extracorporeal membrane oxygenation (ECMO) program were initiated in 2011 and 2013. First, the association of LFD per minute with the 90-day mortality and neurological outcome was analyzed using multiple logistic regression analysis. Then, the temporal changes in LFD were investigated.

**Results:**

Of 175 study subjects who received ECPR, 117 had IHCA. In the multivariate logistic regression, IHCA patients with shorter LFD experienced significantly increased 90-day survival and favorable neurological outcomes (LFD per minute, 90-day survival: odds ratio [OR] = 0.97, 95% confidence interval [CI] = 0.94–1.00, *P* = 0.032; 90-day favorable neurological outcome: OR = 0.97, 95% CI = 0.94–1.00, *P* = 0.049). In the study period, LFD significantly decreased over time (slope − 5.39 [min/3 years], *P* < 0.0001).

**Conclusion:**

A shorter LFD was associated with increased 90-day survival and favorable neurological outcomes of IHCA patients who received ECPR. The quality improvement in administering ECPR over time, including the RRS program and the ECMO program, appeared to ameliorate clinical outcomes.

## Introduction

Cardiac arrest (CA) is one of the leading causes of death. It is a major public-health issue in the world [1, 2]. In the USA, approximately 600,000 people each year are expected to experience sudden CA [1], and 100,000 are expected to experience out-of-hospital CA (OHCA) in Japan [2]. Minimizing the low-flow duration (LFD), which is defined as the time from the start of the resuscitation process to regaining blood circulation, is imperative to improve survival and neurological functions of patients with CA [3]. In in-hospital CA (IHCA) cases, the rapid response system (RRS) plays a key role in shortening LFD and reducing mortality [4, 5].

Extracorporeal cardiopulmonary resuscitation (ECPR) is a promising rescue strategy that has been used to reduce mortality of refractory CA patients [6, 7]. Debates have ensued regarding the association between short LFD and improved outcomes of CA by administering ECPR [8, 9]. A meta-analysis demonstrated the association between LFD and CA outcomes [8, 9]. However, data that present factors that can affect LFD after ECPR are insufficient [8, 9].

The administration of ECPR requires interdisciplinary collaboration, including RRS, to rescue a CA victim [10, 11]. Therefore, quality improvement in administering ECPR, which can be assessed using the time at each workflow step, is plausible and can contribute to LFD improvement and thus survival from CA. Educational programs such as simulation-based learning, which are a part of the efforts to improve the quality of care in terms of RRS in hospitals, can be related to decreasing LFD in CA cases that were administered ECPR [12]. However, to the best of our knowledge, very few studies have investigated the quality improvement in administering ECPR over time and its association with LFD and CA results.

In the present study, we hypothesized that the quality improvement in efforts to shorten the duration of initiating ECPR had decreased LFD over the last 15 years of experience at an academic tertiary care hospital, which in turn has improved the outcomes of IHCA. We first investigated the association between a decreased LFD and improved neurological outcomes in CA patients who received ECPR in an urban university hospital in Japan. Subsequently, we investigated the trend of LFD over the last 15 years. In addition, further analysis was performed to investigate factors that could have contributed to the improvement in the quality (time) of administering ECPR.

## Materials and methods

### Study setting and patients

This observational study was conducted at Chiba University Hospital, Japan, which has a tertiary acute-care center that includes an emergency department (ED) and surgical/medical intensive care units (ICUs) (22 beds). A team comprising physicians, nurses, and clinical engineers who belong to the Department of Emergency and Critical Care Medicine (ECCM) was in charge not only in both the ED and ICUs but also in the RRS. All IHCA patients were resuscitated and treated by this team, including those who required ECPR.

**Fig. 1:**
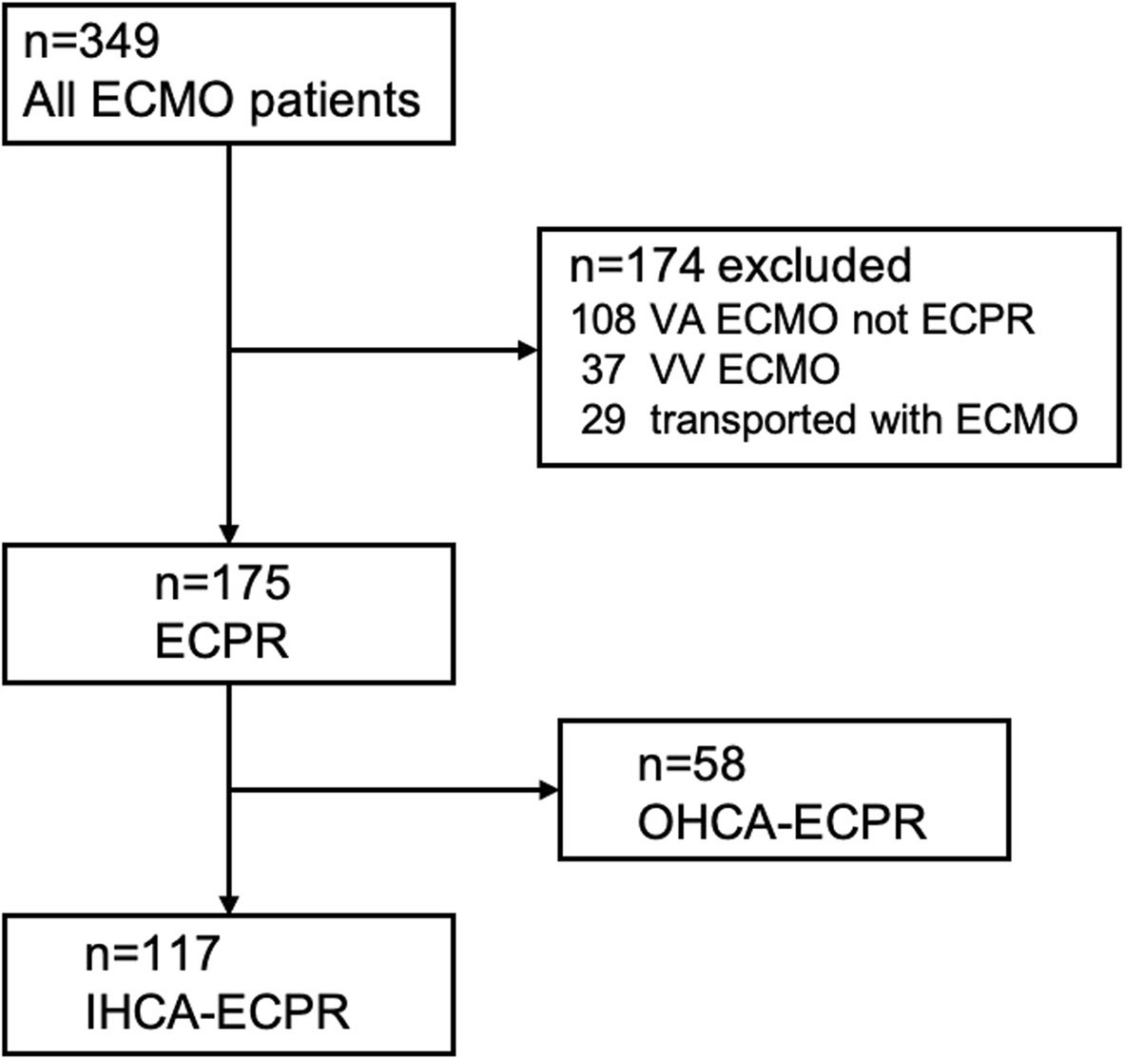
Flow diagram of the study population. In total, 349 ECMO patients were enrolled in the study period from 2003 to 2017. Of 349 ECMO patients, 232 (VA ECMO not ECPR [*n* = 108], VV ECMO [*n* = 37], transported with ECMO [*n* = 29], OHCA-ECPR [*n* = 58]) were excluded, resulting in 117 patients being included in the analysis

All patients who were treated using extracorporeal membrane oxygenation (ECMO) at Chiba University Hospital, Japan, between January 2003 and December 2017 were screened (Fig. 1). Of 349 screened patients, 108 underwent venoarterial (VA)-ECMO with no ECPR, 37 underwent venovenous (VV)-ECMO, and 29 were transported with ECMO administration from outside the hospital. Of 175 ECPR patients, 58 were OHCA patients. These patients were excluded from this study. Finally, 117 IHCA patients who received ECPR were included.

This study was approved by the institutional ethics review board. The review board waived the need for written informed consent from each individual patient.

### ECPR, ECMO, and RRS

The attending physicians in the resuscitation team decided the administration of ECPR based on the following assessment: (1) witnessed status, (2) initial electrocardiogram (ECG), and (3) bystander CPR. The general exclusion criteria included the following: (1) age ≥ 80 years, (2) malignancy, or (3) evidence of severe brain damage.

The cannulation for ECMO treatment was performed by trained physicians in the team. Cannulas that drained the blood from the venous system (21 Fr) and that returned the oxygenated blood to the arterial system (16.5 Fr, CAPIOX (X)®, Terumo, Tokyo, Japan) were inserted in the femoral vein and femoral artery, respectively. The cannula tip in the femoral vein was inserted further to the inferior vena cava and positioned at the right atrium. The cannula position was confirmed by performing either chest x-ray or ultrasound at the bedside. A quick-priming ECMO system (CAPIOX® emergency bypass system, Terumo, Tokyo, Japan) with a membrane oxygenator (CAPIOX (LX)®, Terumo, Tokyo, Japan) was used. During cannulation, ECG and echocardiogram were continuously monitored when available. After performing ECMO, the patients were transferred to the ICU and were provided ECMO support until their hemodynamics stabilized. The ECMO blood flow was controlled according to the condition of the patient’s circulatory and respiratory status. Unfractionated heparin was continuously administered as anticoagulant and adjusted to achieve the targeted activated clotting time of 180 s. The targeted temperature for post-CA patients was maintained between 34 °C and 36 °C for at least 24 h after the onset of CA.

To improve the quality of ECMO administration and RRS, multidisciplinary specialists from the department of ECCM jointly organized the RRS and ECMO programs in 2011, which was revised in 2013. Continuous effort for improvement has been made by committee members comprising five physicians and nine nurses/clinical engineers for the RRS program and seven physicians and nine nurses/clinical engineers for the ECMO program. Educational sessions, including lectures and simulation-based learning, have been conducted regularly at least every 6 months since the programs were established.

The RRS program included the following: (1) establishment of a medical emergency team comprising physicians from the Department of ECCM and nurses/clinical engineers from the ICU, (2) conducting RRS campaigns for hospital staff for educational purposes about the importance of early RRS activation, (3) conducting regular classes of simulation-based training of RRS activation for the floor and unit staff, and (4) introducing a rapid information-sharing system for the RRS team using a texting application that is compliant with the standard for sensitive patient data protection. Medical care from RRS was made available to anyone (patients, visitors, employees, etc.) who required immediate medical attention within the hospital grounds. All hospital staff could activate the RRS code in response to pre-defined criteria [13, 14].

The ECMO program included the following: (1) establishing an ECMO team comprising physicians from the Department of Emergency and Critical Care Medicine and nurses/clinical engineers from the ICU who performed ECMO management; (2) conducting regular educational sessions for all physicians/nurses/clinical engineers, including lecture, water drill, and scenario-based simulation training; (3) engaging in mortality and morbidity conference for ECMO; and (4) optimizing the ECPR procedure.

The RRS team prepared RRS kits, and the ECMO team set up an ECMO cart and an ECMO kit that contained all instruments required for performing ECMO, such as cannulae, portable ultrasound machine, and surgical devices.

### Definition and data collection

Shockable rhythm is defined as ventricular fibrillation or pulseless ventricular tachycardia. No-flow duration (NFD) is defined as the interval from collapse to the first resuscitation (basic or advanced life support). LFD is defined as the interval from the start of CPR to ECMO administration. Cannulation time is defined as the time from performing ECPR by the attending physician to the time when blood circulation in the patient is established using ECMO.

A 90-day favorable neurological outcome is defined as cerebral performance category (CPC) 1–2 [15, 16]. The outcomes were individually evaluated by investigators who were not involved in caring for the patients. Because of the small sample size of the annual data, we combined 3-year data between 2003 and 2017 (15 years), obtained five temporal-group data, and evaluated the temporal changes.

### Statistical analysis

The events per variable (EPV) criterion, especially EPV ≥ 10, were used to determine the minimum required sample size and maximum number of candidate predictors that could be examined. The primary outcome variable was the 90-day survival. The secondary outcome variable was the 90-day neurological outcome. We first performed a multiple logistic regression analysis to assess the differences in the 90-day outcomes using LFD per minute. The analysis was performed to adjust the baseline imbalances of potential confounders (age, initial shockable rhythm, witnessed arrest). Next, the primary analysis of the study was conducted, which assessed temporal changes in LFD. The difference in LFD owing to the initiation site was further analyzed. Two-tailed *P* values < 0.05 were considered significant. The odds ratios (ORs) and 95% confidence intervals (CIs) were provided. Analyses were performed using the JMP Pro (JMP, version 13, SAS, Cary, NC, USA) statistical software.

## Results

Among 117 IHCA patients who underwent ECPR, the median age was 66 years old, 65.8% were male, and the most common initial waveform at cardiac arrest was PEA (65.2%), followed by shockable rhythm (25.7%) and asystole (8.9%) (Table 1). Since it occurred in the hospital, 95.7% of all cases had witness, and bystander CPR was done in all cases. The median LFD was 27 min, and the location of ECPR (where the catheter was inserted) was most common in ICU (38.5%), followed by catheter lab (22.2%), general ward (20.5%), and ER (10.3%). The causes of cardiac arrest were heart diseases 68.4%, hemorrhagic shock 11.1%, septic shock 3.4%, pulmonary embolism 3.4%, respiratory failure 2.6%, and the rest were anaphylactic shock, hyperkalemia, hypothermia, malignant hyperthermia, etc. The 90-day survival rate was 38.8%, and CPC1-2 at 90 days was 31.9% (Table 1).

**Table 1:** Baseline characteristics and outcomes

	Total 117 patients
Baseline characteristics	
Age (years)	66 (46–75)
Male [*n* (%)]	77 (65.8)
Initial rhythm [*n* (%)]	
Shockable rhythm	29 (25.7)
PEA	73 (65.2)
Asystole	10 (8.9)
Any shockable rhythm^a^ [*n* (%)]	53 (50.0)
Witnessed [*n* (%)]	112 (95.7)
Bystander CPR [*n* (%)]	117 (100)
NFD (min)	0 (0–0)
LFD (min)	27 (19–40)
< 20 min [*n* (%)]	28 (26.9)
20–39 min [*n* (%)]	48 (46.2)
Over 40 min [*n* (%)]	28 (26.9)
Time zone [*n* (%)]	
9:00–16:59	47 (41.2)
17:00–0:59	34 (29.8)
1:00–8:59	33 (29.0)
Weekday [*n* (%)]	93 (80.2)
Initiation site [*n* (%)]	
ICU	45 (38.5)
Catheter laboratory	26 (22.2)
General ward	24 (20.5)
ER	12 (10.3)
Operating room	6 (5.1)
Imaging laboratory	3 (2.6)
Outpatient units	1 (0.85)
Cause of cardiac arrest [*n* (%)]	
Heart disease	80 (68.4)
Hemorrhagic shock	13 (11.1)
Septic shock	4 (3.4)
Pulmonary embolism	4 (3.4)
Respiratory failure	3 (2.6)
Other	13 (11.1)
Outcomes	
90-day period	
Survival [*n* (%)]	45 (38.8)
CPC 1 or 2 [*n* (%)]	37 (31.9)
CPC 3–5 [*n* (%)]	79 (68.1)
Length of ECMO (day)	4 (2–6)
Length of ICU stay (day)	10 (4–19)

*CPR* cardiopulmonary resuscitation, *ICU* intensive care unit, *ECMO* extracorporeal membrane oxygenation, *CPC* Cerebral performance category

^a^Any shockable rhythm during CPR

Data are median (interquartile range) for continuous variables

*P* values are calculated using the Mann-Whitney *U* and Fisher exact tests

In the multivariate logistic regression adjustment for baseline characteristics, a shorter LFD was significantly associated with increased 90-day survival and favorable neurological outcomes (LFD per minute, 90-day survival: OR = 0.97, 95% CI = 0.94–1.00, *P* = 0.032; 90-day favorable neurological outcome: OR = 0.97, 95% CI = 0.94–1.00, *P* = 0.049) (Table 2).

**Table 2:** Multivariate logistic regression analysis

	OR (95% CI)	*P*
A. 90-day survival		
Initial shockable rhythm	4.45 (1.64–12.1)	0.0034
Witnessed arrest	0.14 (0.012–1.68)	0.12
Low-flow duration (per minute)	0.97 (0.94–1.00)	0.032
B. 90-day favorable neurological outcome (CPC 1-2)		
Initial shockable rhythm	3.21 (1.23–8.40)	0.017
Witnessed arrest	0.86 (0.11–6.68)	0.88
Low-flow duration (per minute)	0.97 (0.94–1.00)	0.049

*CPC* Cerebral performance category

*P* values are calculated using the multivariate logistic regression analysis

**Fig. 2:**
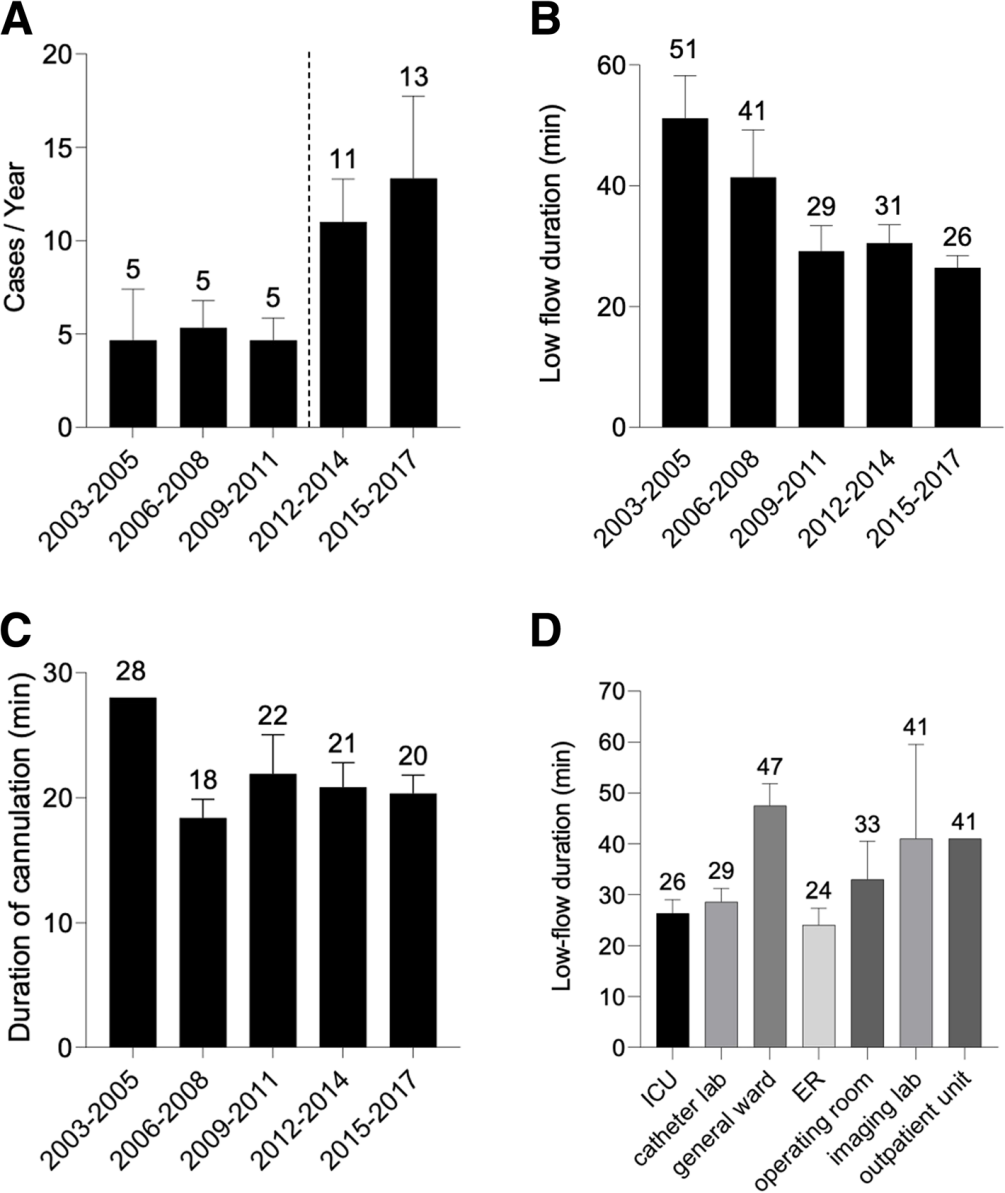
**a** Changes in the annual number of IHCA patients treated with ECPR. The dotted line indicates the time when RRS is introduced. The number of IHCA–ECPR significantly increased by 2.4-fold after the RRS introduction (before RRS [2003–2011] vs. after RRS [2012–2017], 4.9 ± 1.4 vs. 12 ± 1.7 cases/year, *P* = 0.005). The error bars indicate the SEM. **b** Change in LFD over time. LFD in the IHCA–ECPR significantly decreased over time in the study period (slope = −5.39 [min/3 years], *P* < 0.0001). The error bars indicate the SEM. **c** Change in the duration of cannulation over time. The duration of cannulation was not shortened over time (slope = − 0.11 [min/3 years], *P* = 0.90). The error bars indicate the SEM. **d** LFD according to the cannulation location. LFD was shorter in the ICU, catheter laboratory, and ER than that in the general ward, imaging laboratory, and outpatient unit. The numbers of cases are 45 (ICU), 26 (catheter laboratory), 24 (general ward), 12 (ER), 6 (operating room), 3 (imaging laboratory), and 1 (outpatient unit). The error bars indicate the SEM

The annual number of IHCA patients who received ECPR significantly increased by 2.4-fold after the introduction of RRS (before RRS [2003–2011] vs. after RRS [2012–2017], 4.9 ± 1.4 vs. 12 ± 1.7 case/year, *P* = 0.005) (Fig. 2a). LFD significantly decreased over time in the study period (IHCA: slope = − 5.39 [min/3 years], *P* < 0.0001) (Fig. 2b). The cannulation duration did not change significantly in the study period (slope = − 0.11 [min/3 years], *P* = 0.90) (Fig. 2c). LFD was shorter in the initiation sites in the ICU, catheter laboratory, and emergency room (ER) than in the general ward (Fig. 2d).

## Discussion

In this ECPR study, shorter LFD was significantly associated with increased 90-day survival and favorable neurological outcome. IHCA patients who received ECPR significantly increased after the introduction of RRS. LFD was significantly shortened over time during the 15-year study period. The duration of cannulation was not changed.

In a previous single-center study that included 199 IHCA patients who received ECPR, the shorter duration from collapse to ECMO was significantly associated with increased in-hospital survival (hazard ratio = 1.02, *P* < 0.01) [17]. In addition, from the systematic review of IHCA patients who received ECPR (six studies, 570 patients), the survivors had significantly shorter LFD than the non-survivors (survivors vs. non-survivors, 28.7 vs. 46.1 min, *P* < 0.0001) [8]. Our findings were consistent with these results, and in our study, IHCA patients with shorter LFD experienced significantly increased 90-day survival and favorable neurological outcome even after the adjustment for baseline imbalances. These data supported the conclusion that shortening LFD positively affected the survival of IHCA patients.

One of the major findings of this study was that LFD significantly decreased over time during the 15-year study period (reduced rate [min/3 years]; IHCA = − 5.39) (Fig. 1b). Regarding IHCA, RRS played a prominent role in this improvement. The RRS organization was formally accepted in our institution in 2011. This change enabled specialized providers from the Department of ECCM to take over patient care at the bedside and start procedures without asking permission from healthcare providers in other departments; thus, the primary medical problems of patients were immediately treated. This process could have contributed to the increase in the number of IHCA–ECPR over time (Fig. 1a). Additionally, the ECMO training program, which started in 2013, was introduced to all RRS providers, including physicians, nurses, and clinical engineers, which enabled quick initiation of ECPR. The program included rapid and safe initiation approach using guidewire and cannula placement verified by chest x-ray. In line with these initiatives, LFD in IHCA patients decreased from more than 40–50 (2003–2008) to approximately 30 min (2009–2017) (Fig. 2b).

Long-term attempts to improve the outcome of ECPR patients have been reported by pediatricians. An increase in the number of cases and decreased LFD (median 33 min, *P* < 0.001) were demonstrated over time from 1995 to 2008 in Boston Children’s Hospital [18]. A rapid response ECMO (RR-ECMO) program was also reported, which contained four features: (1) introduction of a batch paging system for notification to ECMO team members, (2) training of respiratory therapists to initiate ECMO in the absence of perfusionists, (3) placement of surgical cannulation carts in all pediatric ICUs, and (4) maintenance of crystalloid-primed ECMO circuits dedicated to rapid deployment, which was introduced in 2008 [19]. Before and after the program introduction, complications in the central nervous system significantly decreased over time (OR = 0.48, *P* = 0.04). However, LFD and overall mortality in this study did not show a difference (68 vs. 51 min, *P* = 0.32; OR = 0.99, *P* = 0.99, respectively) [19].

Our program shared many similarities with the RR-ECMO program. Instead of pagers, our RRS adopted a broad intra-network service to share information about ECPR cases that enabled providers to deliver more detailed real-time information. Also, we developed a handy ECPR cart, which helped us to start the ECPR procedure anywhere in the hospital even without any preparation, such as in the endoscopy examination room or in-hospital clinics. Moreover, we prepared an ECPR kit that contained the necessary equipment (forceps, syringes, etc.) for starting ECPR so that anyone in RRS could act as a perfusionist. Many investigators discussed the shortage of staff, such as perfusionists and scrub nurses, especially at nighttime or weekends, because this was the rate-limiting factor for quickly starting ECMO. In our department, all nurses with > 3 years of experience (> 60% of all nurses) participate in simulation-based training as a perfusionist and/or scrub nurse. Because they are always included in, the RRS team in our hospital is always ready to start ECPR without waiting for additional staff. These additional features were considered to have provided further beneficial effects on LFD efficiency and improvement in outcomes.

In this analysis, LFD was found different depending on the location of ECPR, which in the general ward was longer compared to those in the ICU, catheter lab, and ER. Regarding the place of ECPR, there are two strategies performing at the site of origin without moving the patient or transporting the patient to a predetermined location such as ICU, catheter lab, or ER. The advantage of the former is eliminating transport time, while the disadvantage is difficulty in the procedure due to the limitation of necessary space and items. Besides, when performed in the catheter lab, it is advantageous in terms of safety that the guidewire and the tip of the cannula can be checked in real-time. According to time distance, we decide the place of ECPR to minimize LFD, since general wards in the hospital are distant from ICU, catheter lab, and ER. To eliminate time spent searching for items, we set up a handy ECMO cart and ECMO kit that contained all instruments. To ensure the safety of ECPR at unideal sites, we routinely use a portable ultrasound during puncture, which is brought in the ECMO kit, and check wires and cannula tips with portable x-rays. The place of ECPR initiation should be considered depending on the environment of each facility. Still, these efforts allow ECPR to be undertaken quickly and safely anywhere in the hospital.

This study had some limitations. First, this was a retrospective study at a single center. Second, the sample size of the study was not large. Further studies using a larger sample from multiple centers may enhance our study results.

## Conclusions

A shorter LFD was associated with increased 90-day survival and favorable neurological outcomes in IHCA patients who received ECPR. The quality improvement from administering ECPR, including the RRS program and the ECMO program, appeared to ameliorate the clinical outcomes over time.

## Data Availability

The datasets used and/or analyzed in the current study are available from the corresponding author upon reasonable request.

## Abbreviations

CA: Cardiac arrest
CPC: Cerebral performance category
ECCM: Emergency and Critical Care Medicine
ECG: Electrocardiogram
ECMO: Extracorporeal membrane oxygenation
ECPR: Extracorporeal cardiopulmonary resuscitation
ED: Emergency department
ER: Emergency room
ICU: Intensive care unit
IHCA: In-hospital cardiac arrest
LFD: Low-flow duration
NFD: No-flow duration
OHCA: Out-of-hospital cardiac arrest
RR-ECMO: Rapid response- extracorporeal membrane oxygenation
RRS: Rapid response system
VA-ECMO: Venoarterial-extracorporeal membrane oxygenation
VV-ECMO: Venovenous-extracorporeal membrane oxygenation
